# High-sensitivity detection of cryptic *Wolbachia* in the African tsetse fly (*Glossina* spp.)

**DOI:** 10.1186/s12866-018-1291-8

**Published:** 2018-11-23

**Authors:** Daniela I Schneider, Andrew G Parker, Adly M Abd-alla, Wolfgang J Miller

**Affiliations:** 1 Department Cell and Developmental Biology, Center for Anatomy and Cell Biology, Medical University of Vienna, Vienna, Austria; 2 Present Address: Department of Epidemiology of Microbial Diseases, Yale School of Public Health, New Haven, CT USA; 3 Insect Pest Control Laboratory, Joint FAO/IAEA Division of Nuclear Techniques in Food and Agriculture, Vienna, Austria

**Keywords:** *Wolbachia*, Low-titer symbiont detection limit, Stellaris® fluorescence in situ hybridization, Tissue tropism

## Abstract

**Background:**

In African tsetse flies *Glossina*, spp. detection of bacterial symbionts such as *Wolbachia* is challenging since their prevalence and distribution are patchy, and natural symbiont titers can range at levels far below detection limit of standard molecular techniques. Reliable estimation of symbiont infection frequency, especially with regard to interrelations between symbionts and their potential impact on host biology, is of pivotal interest in the context of future applications for the control and eradication of *Glossina*-vectored African trypanosomosis. The presence or absence of symbionts is routinely screened with endpoint polymerase chain reaction (PCR), which has numerous advantages, but reaches its limits, when detecting infections at natural low titer. To not only determine presence of native tsetse symbionts but also to localize them to specific host tissues, fluorescence in situ hybridization (FISH) can be applied. However, classic FISH assays may not detect low-titer infections due to limitations in sensitivity.

**Results:**

We have compared classic endpoint PCR with high-sensitivity blot-PCR. We demonstrate that the latter technique allows for clear detection of low-titer *Wolbachia* in the *morsitans* and *palpalis* groups while classic endpoint PCR does not. In order to localize *Wolbachia* in situ in high and low-titer *Glossina* species, we applied high-end Stellaris® *r*RNA-FISH. We show that with this high sensitivity method, even low amounts of *Wolbachia* can be traced in specific tissues. Furthermore, we highlight that more tissues and organs than previously recorded are infested with *Wolbachia* in subspecies of the *morsitans* and *palpalis* groups.

**Conclusions:**

Our results demonstrate that overall symbiont infection frequencies as well as the presence in specific host tissues may be underestimated when using low-sensitivity methods. To better understand the complex interrelation of tsetse flies and their native symbionts plus the pathogenic trypanosomes, it is important to consider application of a broader range of high-sensitivity detection tools.

**Electronic supplementary material:**

The online version of this article (10.1186/s12866-018-1291-8) contains supplementary material, which is available to authorized users.

## Background

In the African tsetse fly (*Glossina* spp., Diptera: Glossinidae), detection of bacterial symbionts such as *Wolbachia* is challenging since their prevalence and distribution are patchy [1], and their natural titers can range at levels far below detection limit of standard molecular techniques [2, 3]. Reliable estimation of symbiont infection frequency, however, especially with regard to interrelations between the symbionts and their potential impact on host biology, is of pivotal interest in order to control and eradicate African trypanosomosis in the future [4]. While classic endpoint PCR has numerous advantages, it soon reaches its limits, when it comes to detecting very low loads of symbionts (low-titer infections), and localize the symbionts to specific host tissues (tissue tropism). Classic fluorescence in situ hybridization (FISH) using *16S r*RNA oligo probes is the method of choice for following tissue tropism. However, sensitivity may be too low for symbiont detection in biologically relevant host organs like testes but also for the in situ detection of generally low-titer infections such as the ones of *G. p. gambiensis*. In the presented study, we have compared the feasibility of classic PCR and blot-PCR for low-titer *Wolbachia* in species and subspecies of the genus *Glossina*. Furthermore, we took advantage of the novel Stellaris® technology to trace such low-titer *Wolbachia* in situ. As known from recent studies, not only high-titer infections, but also *Wolbachia* low-titer infections impact host biology [5]. Hence, their reliable detection with particular regard to their location in situ, is important for better understanding host-symbiont relations plus the crosstalk with trypanosome parasites.

## Results

### Low-titer *Wolbachia* infections in *Glossina* spp. may be overlooked with standard endpoint-PCR techniques

To demonstrate the advantage of more sensitive detection methods over standard techniques, we employed and compared two endpoint PCR techniques plus one high-end blot-PCR. First, we tested the classic *Wolbachia* marker *wsp* (Wolbachia outer surface protein gene) with *Glossina* subspecies from the *palpalis* and *morsitans* groups (Table 1).

**Table 1:** List of G*lossina* and *Drosophila* strains analyzed in this study

	Species	Species group	Strain/abbr.	Origin/reference				
D*rosophila* strains	*Drosophila simulans*	*simulans*	NouméaTC	[18]	*Wolbachia* negative control			
	*Drosophila willistoni*	*willistoni*	P98	[19]	*Wolbachia* positive control			
					*Wolbachia* status (experimental)			
					*wsp*	*ARM*	blot	FISH
*Glossina* strains	*Glossina morsitans morsitans*	*morsitans*	*Gmm*	Takáč Lab, Slovak Academy of Sciences, Bratislava, Slovakia	+++	+++	+++	+++
	*Glossina morsitans centralis*	*morsitans*	*Gmc*	Insect Pest Control Laboratory FAO/IAEA, Vienna, Austria	+++	+++	+++	+++
	*Glossina swynnertoni*	*morsitans*	*Gsw*	Insect Pest Control Laboratory FAO/IAEA, Vienna, Austria	–	++	++	*nd*
	*Glossina palpalis palpalis*	*palpalis*	*Gpp*	Insect Pest Control Laboratory FAO/IAEA, Vienna, Austria	–	–	–	–
	*Glossina palpalis gambiensis*	*palpalis*	*Gpg*	Insect Pest Control Laboratory FAO/IAEA, Vienna, Austria	–	–	+	+
	*Glossina fuscipes fuscipes*	*palpalis*	*Gfu*	Insect Pest Control Laboratory FAO/IAEA, Vienna, Austria	–	–	+?	*nd*

Two *Drosophila* strains were used as *Wolbachia*-positive and -negative controls (P98, NouméaTC). The table lists all *Glossina* strains used for experiments, including *Wolbachia* infection status based on PCR (Wolbachia outer surface protein gene, Wolbachia A-supergroup repeat motif,); blot-PCR using a *wsp* probe (listed as ‘blot’), and fluorescence in situ hybridization with *Wolbachia 16*-*23S r*RNA probe. *Wolbachia* infection titer is indicated by ‘+’ (low), ‘++’ (intermediate), and ‘+++’ (high). Abbreviations: *wsp* Wolbachia outer surface protein gene, *ARM* Wolbachia A-supergroup repeat motif, FISH fluorescence in situ hybridization, *nd* not determined

**Fig. 1:**
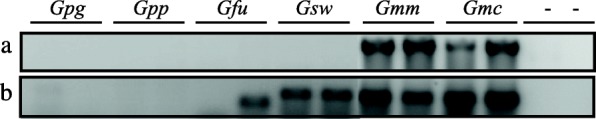
Detection of *Wolbachia* in *Glossina* females and males via polymerase chain reaction (PCR). **a** *Wolbachia*-specific single copy *wsp*-PCR detects the symbiont only in high-titer *Gmm* (♀, ♂) and *Gmc* (♀, ♂). **b** The more sensitive multicopy *ARM*-PCR additionally detects *w*Gsw (*Wolbachia* of *G. swynnertoni*). In the *Wolbachia*-uninfected *G. f. fuscipes*, *ARM*-PCR amplifies a possibly nonspecific product of smaller size. Negative controls are *Wolbachia*-uninfected *Drosophila simulans*, NouméaTC (*Dsim*^minus^) and non-template control (NTC). Females are first on gel. Each PCR was at least repeated once in order to confirm results. Abbreviations: *wsp* Wolbachia outer surface protein gene

Endpoint PCR using the single copy *wsp* marker clearly detects high-titer *Wolbachia* in females and males of *G. m. morsitans* (*Gmm*) and *G. m. centralis* (*Gmc*), but *G. swynnertoni* (*Gsw*) and *G. p. gambiensis* (*Gpg*) seem uninfected. We did also not detect *Wolbachia* in the two subspecies *G. p. palpalis* (*Gpp*) and *G. f. fuscipes* (*Gfu*)*,* which were previously reported *Wolbachia*-negative (Fig. 1a) [1]. Next, we applied the multicopy locus *ARM* (Wolbachia A-supergroup repeat motif) [3] to the same sample set (Fig. 1b). Similar to *wsp*-PCR, the more sensitive multicopy specific *ARM*-PCR easily detects high-titer *Wolbachia* of *Gmm* and *Gmc*. In addition, it traces the earlier described low-titer infection in *Gsw* [3]. Interestingly *ARM*-PCR successfully amplifies a smaller fragment in *Gfu* males but not females, (Fig. 1b; and see below).

### High-end blot-PCR increases *Wolbachia* detection limit

**Fig. 2:**
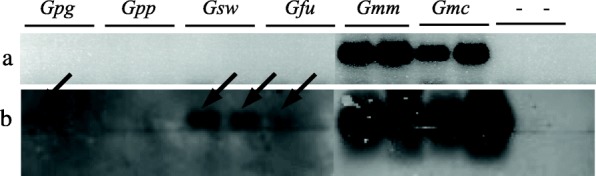
Detection of *Wolbachia* in *Glossina* group females and males via combined PCR-hybridization (blot-PCR). **a** *wsp*-PCR before hybridization only detects high-titer *w*Gmm and *w*Gmc (♀, ♂ for both). **b** Hybridization with a digoxygenin-labeled *wsp*-probe detects *Wolbachia* in *G. p. gambiensis* females. *G. swynnertoni* females and males are also positive for *Wolbachia* upon hybridization, and even the *Wolbachia*-uninfected *G. fuscipes* shows a slight band after hybridization, suggesting potential presence of an ultra low-titer infection (♀). Negative controls are *Wolbachia*-uninfected *Drosophila simulans*, NouméaTC (*Dsim*^minus^) and non-template control (NTC). Each PCR and consequent blotting was at least repeated once in order to confirm results. Abbreviations: *wsp* Wolbachia outer surface protein gene

As demonstrated above, *wsp* and *ARM* markers are efficient tools to screen for *Wolbachia* infection status when symbiont titers are at high or medium levels. In order to further increase the detection limit of our assay, we employed a combined PCR-hybridization (blot-PCR) assay [2, 6]. This method has proven reliable in detecting low loads of *Wolbachia*, which were undetected in standard PCR assays such as the *wsp*-based PCR [2, 5, 6]. As shown in Fig. 2b consequent hybridization with an internal *Wolbachia*-specific *wsp*-probe allows more *Wolbachia* to be traced than in the *wsp*-PCR assay shown in Fig. 2a. Similar to classic endpoint *wsp*-PCR, blot-PCR detects high-titer *Wolbachia* of *Gmm* and *Gmc* (even before hybridization; shown in Fig. 2a). It further allows detection of the symbiont in *Gpg* females (Fig. 2b, black arrow), and both sexes of *Gsw* (Fig. 2b, black arrows). Also, *Gfu*, which was reported *Wolbachia*-uninfected previously [1], seems to amplify a weak *wsp*-band on the blot (Fig. 2b, black arrow), although it is very faint and its reliability should thus be questioned.

### High-end Stellaris® rRNA-FISH helps to detect low-titer *Wolbachia* infections in situ

**Fig. 3:**
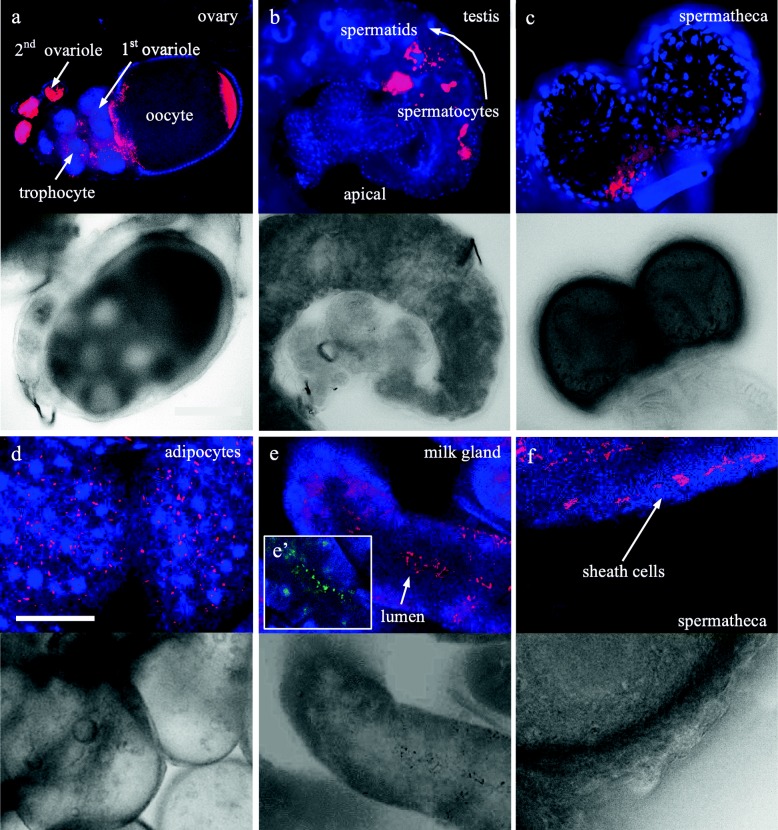
Stellaris® *r*RNA-FISH on high-titer *Glossina* species (*G. m. morsitans*, *G. m. centralis*). **a** In the ovary *Wolbachia* are present in the first and in the second ovariole plus at the posterior pole of the oocyte. **b** *Wolbachia* are not detected in early stages of spermatogenesis (hub cells, spermatogonia) but are visible in later stages (spermatocytes, spermatids). Also the spermatheca, particularly the sheath cells (**c**, **f**), as well as adipocytes (**d**) and milk gland (**e**) are infected with *Wolbachia*. (**e**’) *Wigglesworthia*, primary symbiont of *Glossina*, are also present in the milk gland. *Wolbachia* are stained in pink (*16*-*23S* *r*RNA), *Wigglesworthia* are shown in green (*16*-*23S* *r*RNA), *Glossina* DNA is stained in blue (4′,6-diamidino-2-phenylindole), brightfield images for better orientation are presented for a-f. Scale bar is 100 μm

In order to localize *Wolbachia* in situ, we tested various *Glossina* species via fluorescence in situ hybridization (FISH). As standard FISH is not sensitive enough to detect low-titer *Wolbachia* (Schneider, *unpublished*) we employed high-end Stellaris® *r*RNA-FISH. This method uses a set of up to 48 oligonucleotides, which collectively bind along the same target transcript (see Methods section for probe design). First, we tested in situ localization of high-titer *Wolbachia* in *Gmm* and *Gmc* (Fig. 3). As shown in Fig. 3, the symbionts are present in the reproductive organs of females and males (Fig. 3a, b) plus in the sheath cells of the spermatheca (Fig. 3c, f) in both high titer hosts. *Wolbachia* also infect adipocytes, the main fat body cells (Fig. 3d), and the milk glands (Fig. 3e). The latter tissue also houses tsetse’s primary symbiont *Wigglesworhtia*, as shown via costaining with symbiont-specific *16S* probe (Fig. 3e’).

**Fig. 4:**
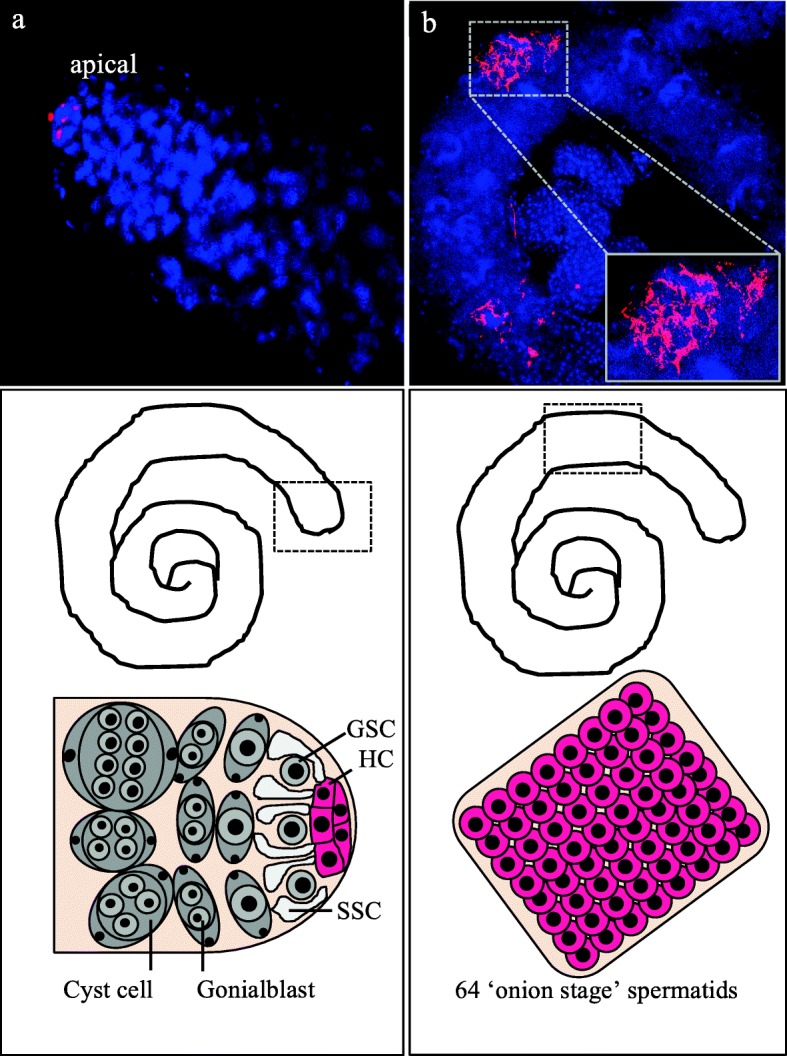
Stellaris® *r*RNA-FISH on testes of high-titer *G. m. morsitans*. **a** *Wolbachia* accumulating in the hub cells of the apical part (indicated by dotted box) of the testis. **b** In later stages of spermatogenesis, *Wolbachia* are present in 64 ‘onion stage’ spermatids. *Wolbachia* are stained in pink (*16*-*23S r*RNA), *Glossina* DNA is stained in blue (4′,6-diamidino-2-phenylindole). Minimum three individuals from each sex were dissected and processed for FISH. Abbreviations: GSC germline stem cells, SSC somatic stem cells, HC hub cells

So far, the presence of *Wolbachia* in male gonads of *Glossina* was never reported in the literature [7] although *Wolbachia* from *Gmm* and *Gmc* are well known for causing strong cytoplasmic incompatibility (CI) [2, 8]. By using high-end Stellaris® *r*RNA-FISH, however, we clearly detected the symbiont in the testes of both *Gmm* and *Gmc.* Most interestingly, we did not observe overall infection of the reproductive organ, but a strong restriction to certain stages of spermatogenesis (Fig. 4). We detected *Wolbachia* in the hub of the testis in *Gmm*, which is formed by non-dividing stromal cells connected to the surrounding stem cells (Fig. 4a). Further, *Wolbachia* are present in a later stage of spermatogenesis, the so-called 64 ‘onion-stage’ spermatids (Fig. 4b).

**Fig. 5:**
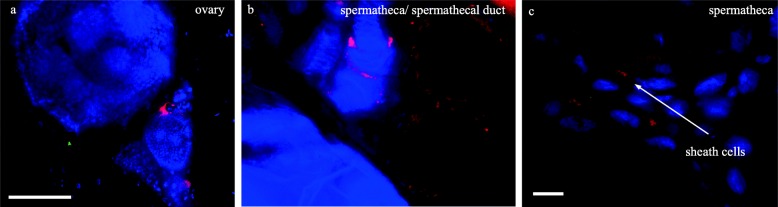
Stellaris® *r*RNA-FISH on low-titer *G. p. gambiensis* (*Gpg*). *Wolbachia* are present in (**a**) ovaries, **b** spermatheca, spermathecal duct, and (**c**) in the sheath cells of the spermatheca. *Wolbachia* are stained in pink (*16*-*23S r*RNA), *Glossina* DNA is stained in blue (4′,6-diamidino-2-phenylindole). Scale bars are 100 μm (**a, b**) and 10 μm (**c**)

We further analyzed in situ localization of *Wolbachia* in the low-titer species *G. p. gambiensis* (*Gpg*)*,* and could clearly detect the symbiont in ovaries (Fig. 5a). Similarly, to the situation in the high-titer species *Gmm* and *Gmc*, *Wolbachia* was localized to the sheath cells of the spermatheca (Fig. 5c). Besides that, *Wolbachia* are also present in the spermathecal duct (Fig. 5b, c). So far, we were not able to elucidate the presence of *Wolbachia* in males of low-titer *Glossina* species unambiguously.

## Discussion

Detection of *Wolbachia* inside the host can be challenging when symbiont loads are low. In such cases, detection techniques of higher sensitivity are required to avoid false negative typing of specimens. We have compared in different species and subspecies of *Glossina*, the feasibility of single copy *Wolbachia*-specific PCR (*wsp*) with an improved multicopy locus assay (*ARM*). The standard Wolbachia outer surface protein gene (*wsp*) PCR is commonly used to detect and type *Wolbachia* in various host species, but has the disadvantage of low detection limit when it comes to low-titer infections. For the tsetse fly samples where standard *wsp-*PCR was not sufficient to detect *Wolbachia,* the more sensitive *ARM*-marker, however, enhanced the detection sensitivity and thus detected low-titer *Wolbachia* infection in *Gsw.* This finding is a confirmation of previous results on the feasibility of *ARM* for low-titer *Wolbachia* infections [3]. However, *Wolbachia* of *Gpg*, *Gpp* and *Gpal* could not be traced with *ARM* in laboratory-reared flies, although the presence of *Wolbachia* in these species was reported previously in field collected samples [1]. An explanation for this observation could be the complete lack of the *ARM* motif from the genomes of these *Wolbachia* strains. *Gpg* falls into a different species group than *Gpal*, *Gmm* and *Gmc*, which could account for differences in the associated *Wolbachia* strains, such as presence or absence of a repeat motif. Alternatively, the *Wolbachia* titer could simply be below the detection limit of *ARM*-PCR in these species. Finally, we could not detect any *Wolbachia* in *Gfu* and *Gpp* using *ARM*-PCR, which agrees with a previous study that reported those species uninfected ([1] and references therein). In *Gfu* males, however, we found a smaller *ARM*-fragment, which could be explained by a potential degenerated Y-chromosomal copy of the motif. This assumption though needs to be tested by further sequencing experiments.

We recently reported on the advantage of the multicopy *ARM*-marker over blot-PCR, due to less time consumption (no blotting, hybridization and detection steps) but higher sensitivity, at least for *Drosophila* specimens [2]. Furthermore, the *ARM* multicopy target variant seems to be restricted to A-supergroup *Wolbachia* strains and absent in the others [3]. However, while the previous study focused on *Drosophila*, we report on various *Glossina* subspecies in the present study, and for the tested *Glossina* specimens, *ARM* does not have an advantage over blot-PCR. In contrast, the blot technique confirms an earlier study, which claims an increased sensitivity for *Wolbachia* by up to 1000-fold [6]. As mentioned before, the reason for this might be very low copy number or absence of the *ARM* motif from *Wolbachia* in certain subgroups within the genus *Glossina*.

Most unexpectedly, we detected a weak *Wolbachia* signal in *Gfu* females via blot-PCR. This is interesting as this subgroup was reported uninfected previously [1]. However, it is possible that *Wolbachia* is indeed present in *Gfu*, but in ‘ultra-low’ amounts and is hence very difficult to detect or as a translocated chromosomal copy. The latter event is not unlikely since the presence of multiple extensive chromosomal insertions of *Wolbachia* into the host genome (chrWol) was reported in *Gmm* [1, 9, 10]. Furthermore, there may be a variation in *Wolbachia* infection frequencies between populations of *Gfu*. Hence, larger numbers of individuals, preferentially from lab colonies as well as from different field collections, need to be tested in order to confirm the presence or absence of low-titer *Wolbachia*.

It was highlighted recently that not only the titer of symbionts per se, but especially the localization of them inside the host, is important in the context of interactions between the two [2, 11–13]. Hence, detecting the symbiont’s location in specific tissues and organs of the host is an important aspect of host-symbiont studies. To address this question in *Glossina* subspecies, we employed high-end Stellaris® RNA-FISH. As anticipated we detected massive *Wolbachia* infestation in gonads of high-titer *Gmm* and *Gmc* females. However, we also found *Wolbachia* in lumen and secretory cells of the milk gland of *Gmm*, along with the primary tsetse fly symbiont *Wigglesworthia* (Fig. 3e’). This is in contrast to an earlier report, which showed that the milk gland is infected by the two other tsetse symbionts, *Wigglesworthia* and *Sodalis*, but not by *Wolbachia* [7]. The presence of *Wolbachia* in the milk gland may point towards a yet undiscovered alternative transmission route from mother to offspring, similar to *Wigglesworthia* transmission (reviewed in [14, 15]). Since the developing larva feeds on milk lactated from the milk gland during its intrauterine development, it is tempting to speculate that the *Wolbachia* of this viviparous host system might not only be transmitted trans-ovarially but also nutritionally to the offspring.

Another tissue that was previously reported *Wolbachia*-uninfected is the fat body, mainly composed of adipocyte cells [7]. In contrast to that earlier finding, we clearly detected *Wolbachia* in these cells by using Stellaris® *r*RNA-FISH. Adipocytes play an important role in insect metabolism as they store and release energy in response to the energy demands of the insect [16]. Hence, it is possible that *Wolbachia* found a way to exploit this host-provided energy source for their own purpose. However, this and their milk gland tropism have to be further elucidated in more detailed studies.

As *Wolbachia* are transmitted maternally, the presence of the symbionts in male tsetse flies has not been tested and/or reported so far. However, a recent study demonstrated that *Sodalis*, the second maternally transmitted facultative symbiont of *Glossina*, are transmitted paternally too [17]. In the context of this study, the detection of high-titer *Wolbachia* in *Gmm* and *Gmc* males via FISH is particularly interesting. It pinpoints that *Wolbachia* not only persist in male tsetse flies, but are obviously restricted to certain stages of spermatogenesis in the testis. The importance of such host-derived restriction of the symbiont was recently reported in other systems and tissues [11–13]. Moreover, the detection of *Wolbachia* in the male reproductive organ is important in relation to the capacity of *Wolbachia* strains to CI. This is in accordance with recent reports on the ability of *Wolbachia* from *Gmc* and *Gmm* to trigger CI [2, 8].

Classic FISH is not sufficient to detect low-titer *Wolbachia* in *Glossina* subspecies (data not shown). However, with the help of Stellaris® *r*RNA-FISH technique, we could show that *Wolbachia* are present in the reproductive tissues of *Gpg*. This finding confirms our data from blot-PCR, where we could also detect *Wolbachia* in *Gpg*. The presence of *Wolbachia* in the uterus has not been reported previously [10] and it remains to be elucidated why the symbionts are accumulating in this tissue.

## Conclusion

Here we demonstrate that detection of symbionts inside the host requires various tools. Low-titer infections can only be detected with high-sensitivity methods such as combined PCR and hybridization. Furthermore, we show that high-end Stellaris® technology helps to uncover the localization of *Wolbachia* to tissues that were previously characterized as uninfected. We also highlight how feasible this method is to trace low-titer *Wolbachia* in two *Glossina* subspecies.

Taken together these results demonstrate that choosing a sensitive detection tool for determining symbiont infection status is very important. Gaining deeper insight into the biological and functional interactions between *Glossina* species, their native symbionts and the pathogenic trypanosomes, we should not underestimate their actual infection frequencies and infection localization through applying only low sensitivity detection tools.

## Methods

### Fly strains

In this study, we employed *Glossina* species from the *palpalis* group (*G. palpalis palpalis* - *Gpp, G. palpalis gambiensis* - *Gpg, G. f. fuscipes* - *Gfu*), the *morsitans* group (*morsitans morsitans* - *Gmm*, *G. morsitans centralis* - *Gmc*, *G. pallidipes* - *Gpa*, *G. swynnertoni* - *Gsw*), and from the *fusca* group (*G. brevipalpis* – *Gbr*)*.* The *Wolbachia*-uninfected strain *Drosophila simulans*, NouméaTC [18] served as negative control for experiments. The *Wolbachia-*positive control was *D. willistoni*, Panama98 (P98), carrying native high-titer *Wolbachia* (*w*Wil; [19]). Strains used in this study are summarized in Table 1.

### Detection and quantification of *Wolbachia*

DNA was extracted from pools of 5–15 *Drosophila* or a single adult tsetse fly. Individuals were disrupted with a TissueLyser TL bead mill, and DNA was consequently extracted using Puregene chemistry (Qiagen, Germany). Extracts were stored at − 20 °C until used for experiments. For classic endpoint-PCR, the *Wolbachia*-specific outer surface protein gene (*wsp*) was used to determine infection status. Diagnostic *wsp*-PCR reactions were performed as described in [20]. In addition to the single-copy *wsp* marker, the multi copy *ARM* locus (Wolbachia A-supergroup Repeat Motif) was used to screen for *Wolbachia* infection [3]. Endpoint-PCR combined with hybridization (blot-PCR) was performed as described in [2, 6]. Briefly, *Wolbachia*-specific *wsp*-PCR was run on genomic DNA samples from all three species groups followed by gel electrophoresis on a 1% agarose gel. Amplicons were then transferred on a positively charged nylon membrane via capillary blotting, cross-linked and finally hybridized overnight with a digoxygenin-labeled internal *wsp*-probe. Detection was performed using sheep anti-DIG-conjugate (Roche, Germany).

### High-end Stellaris® *r*RNA-fluorescence in situ hybridization (FISH)

Custom probe sets against *Wolbachia 16S*–*23S* ribosomal RNA transcripts were designed using the Stellaris® FISH probe designer (www.biosearchtech.com/support/tools/design-software/stellaris-probe-designer; Additional file 1: Table S1). Additional probes targeting *16S*–*23S r*RNA transcripts of *Wigglesworthia* and *Sodalis* were designed. We have employed triple staining and FISH on *Wolbachia*-uninfected plus tetracycline-treated flies to test for cross-reaction of probes with each other and with other bacteria (Additional file 2: Figure S1). Tissues for FISH (ovary, spermatheca, uterus, milk gland, testis, gut) were dissected in sterile 1× phosphate-buffered saline (PBS) under a dissection microscope, stored on ice until enough material was collected and finally washed with ice-cold 1× PBS. Tissues were fixed (3.7% formaldehyde, 1× PBS, 0.15% Triton-X 100; nuclease-free water) for 20 min at room temperature followed by three washes for 10 min each in 1× PBS with 0.03% Triton-X 100 (PBS-T). PBS-T was replaced by absolute ethanol and samples were permeabilized overnight under constant agitation at 4 °C. Tissues were washed for 5 min with washing buffer (2× saline sodium citrate [SSC], 10% deionized formamide, nuclease-free water) at room temperature and then hybridized overnight at 37 °C in 50 μl hybridization mix (0.1% dextrane sulfate, 2× SSC, 10% deionized formamide, nuclease-free water and 2.5–3 pmol of each Stellaris® DNA-probe (Biosearch Technologies, USA). *Wolbachia* probe is described in detail in Additional file 1: Table S1. Post-hybridization, tissues were quickly rinsed in washing buffer at room temp (2 × 5 min), followed by two washes for 30 min each at 37 °C. After washing, tissues were equilibrated in 1× PBS with 0.01% Triton-X 100 for 5 min at room temperature and then incubated with Alexa Fluor® 488 phalloidin (Invitrogen, USA) recognizing F-actin (1:100 in 1× PBS-T) for 1.5 h. After one quick washing step with 1× PBS-T, tissues were stained with 4′,6-diamidino-2-phenylindole (0.2 μg/ml in 2× SSC) for 10 min at room temperature, washed 2× with 1× SSC and finally mounted in Roti-Mount© (Carl Roth, Germany) on sterile slides. Visualization of hybridized tissues was performed on either a Nikon A1 or an Olympus Fluoview 3000 confocal microscope.

## Additional files

Additional file 1: **Table S1.** Sequences and positions of the 48 oligos within the *Wolbachia 16S* and *23S* ribosomal RNA used for Stellaris® *r*RNA-FISH. (PDF 90 kb)

Additional file 2: **Figure S1.** Control FISH on *Glossina spp*. (a) *G. m. morsitans* ovary hybridized with *Wolbachia* (red), and *Wigglesworthia* (green) probes in parallel. *Wigglesworthia* probe does not cross-react with *Wolbachia*. (b) Milk gland of *Wolbachia*-uninfected *G. p. palpalis* hybridized with *Wigglesworthia* (green) and *Wolbachia* (red) probes. While *Wigglesworthia* is recognized in the lumen of the gland, *Wolbachia* probe does not give any signal. (c) Triple staining (*Wolbachia* in red, *Wigglesworthia* in green, *Sodalis* in pink) on tetracyline-treated *G. m. morsitans* female. None of three probes give a signal in the ovary. Fluorophore labels of *16*-*23S* ribosomal RNA probes are FITC (*Wigglesworthia*), CAL Fluor Red 590 (*Wolbachia*), and Quasar 670 (*Sodalis*). *Glossina* DNA is stained in blue (4′,6-diamidino-2-phenylindole). (PDF 31389 kb)

## Abbreviations

*ARM*: Wolbachia A-supergroup repeat motif
FISH: fluorescence in situ hybridization
*Gbr*: *G. brevipalpis*
*Gfu*: *G. f. fuscipes*
*Gmc*: *G. morsitans centralis*
*Gmm*: *G. morsitans morsitans*
*Gpa*: *G. pallidipes*
*Gpg*: *G. palpalis gambiensis*
*Gpp*: *G. palpalis palpalis*
*Gsw*: *G. swynnertoni*
