# Supplementary material for: High-sensitivity detection of cryptic Wolbachia in the African tsetse fly (Glossina spp.)
Source: BMC Microbiol. 2018 Nov 23;18(Suppl 1):140. doi: 10.1186/s12866-018-1291-8 (PMC6251158; doi:10.1186/s12866-018-1291-8)
Supplement: Supplementary file 1 — Table S1. Sequences and positions of the 48 oligos within the Wolbachia 16S and 23S ribosomal RNA used for Stellaris® rRNA-FISH. (PDF 90 kb) [file 12866_2018_1291_MOESM1_ESM.pdf]

Wolbachia 16S-23S rRNA probes			
**Probe number**	**Probe (5' → 3')**	**Probe Position***	**Percent GC**
1	gttcattctgagctaggatc	8-28	45.0
2	ttcgacttgcatgtgttagg	40-60	45.0
3	ctgccactaagttataccat	82-92	40.0
4	ttcctatacattactcaccc	104-124	40.0
5	ccgtttccaacaattattcc	139-159	40.0
6	agggcgtatacggtattagt	161-181	45.0
7	tagcaataaatttttccccc	183-203	35.0
8	tccaccaactagctaatcta	219-239	40.0
9	tagatcattgccttggtagg	246-266	45.0
10	tgatcatcctctcagatcag	269-289	45.0
11	cgtatctcagttccagtgtg	291-311	50.0
12	ttcgcccattgtccaatatt	342-362	40.0
13	ttcttcactcatgcggcatg	375-395	50.0
14	agagctttacaacccaaagg	397-417	45.0
15	cgtcattatcttcctcacta	419-439	40.0
16	gagttagccaggacttcttc	448-468	50.0
17	ttacgcccaataattccgaa	508-528	40.0
18	aacttactaatccgcctacg	534-554	40.0
19	aagcaattccaaggttgagc	571-591	45.0
20	tctatcctctttcaatctct	607-627	45.0
21	atttcacctctacactagga	632-652	40.0
22	ggtgttcctcctaatattta	655-675	35.0
23	gcgtcagatttgaaccagat	691-711	45.0
24	tatctaatcctgtttgctcc	729-759	40.0
25	tttacagcgtggactaccag	751-771	50.0
26	cttcccatatttaacattca	774-794	30.0
27	atgtttaacgcgttagctgt	810-830	40.0
28	gttttaatcttgcgaccgta	843-863	40.0
29	ttttcgcgttgcatcgaatt	910-930	40.0
30	tccatgtcaaggagtggtaa	933-953	45.0
31	ctatcccttcgaataggtat	957-977	40.0
32	tgtgtgaaacccggacgaac	982-1002	55.0
33	acgacacgagctgacgacag	1014-1034	60.0
34	tgcgggacttaacccaacat	1037-1057	50.0
35	aactaaggatgagggttgcg	1062-1082	50.0
36	cttaaagtccccagcattac	1090-1110	45.0
37	cttcctccagtttatcactg	1118-1138	45.0
38	tgatgacttgacatcatccc	1141-1161	45.0
39	tgtgtagcccactccataag	1166-1186	50.0
40	cattgtagccaccattgtag	1190-1210	45.0
41	ttaagggattagcttagcct	1225-1245	40.0
42	acaatccgaactgagatggc	1247-1267	50.0
43	caacttcatgcactcgagtt	1274-1294	45.0
44	gatccacgattactagcgat	1296-1316	45.0
45	cgagaacgtattcaccgtgg	1322-1342	55.0
46	tgacgggcagtgtgtacaag	1345-1365	55.0
47	cgttagcttcgagtgaaacc	1378-1398	50.0
48	ttaaataactccctccttgc	1406-1426	40.0

Table S1. Sequences and positions of 48 oligos within the *Wolbachia 16S* and *23S* ribosomal RNA transcript designed using the Stellaris FISH probe designer (www.biosearchtech.com).
