# Supplementary figures and images for: High-sensitivity detection of cryptic Wolbachia in the African tsetse fly (Glossina spp.)
Source: BMC Microbiol. 2018 Nov 23;18(Suppl 1):140. doi: 10.1186/s12866-018-1291-8 (PMC6251158; doi:10.1186/s12866-018-1291-8)

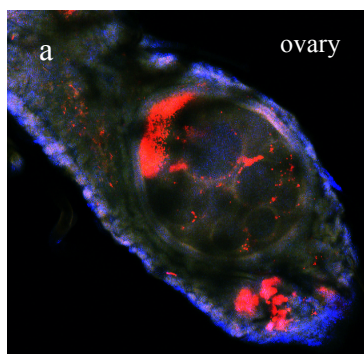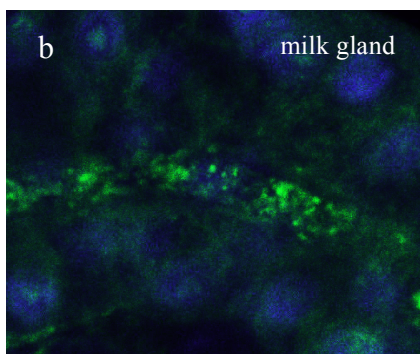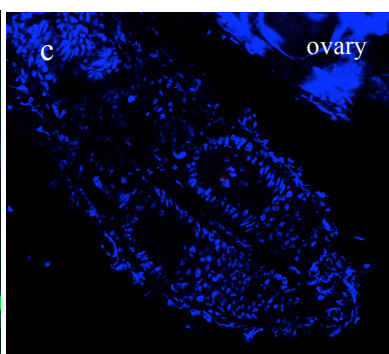

Supplement: Supplementary file 2 — Figure S1. Control FISH on Glossina spp. (a) G. m. morsitans ovary hybridized with Wolbachia (red), and Wigglesworthia (green) probes in parallel. Wigglesworthia probe does not cross-react with Wolbachia. (b) Milk gland of Wolbachia-uninfected G. p. palpalis hybridized with Wigglesworthia (green) and Wolbachia (red) probes. While Wigglesworthia is recognized in the lumen of the gland, Wolbachia probe does not give any signal. (c) Triple staining (Wolbachia in red, Wigglesworthia in green, Sodalis in pink) on tetracyline-treated G. m. morsitans female. None of three probes give a signal in the ovary. Fluorophore labels of 16-23S ribosomal RNA probes are FITC (Wigglesworthia), CAL Fluor Red 590 (Wolbachia), and Quasar 670 (Sodalis). Glossina DNA is stained in blue (4′,6-diamidino-2-phenylindole). (PDF 31389 kb) [file 12866_2018_1291_MOESM2_ESM.pdf]
